# Supplementary material for: A hierarchy of time constants and reliable signal propagation in the marmoset cerebral cortex
Source: Nat Commun. 2025 Nov 26;16:11640. doi: 10.1038/s41467-025-66699-4 (PMC12749727; doi:10.1038/s41467-025-66699-4)
Supplement: Supplementary file 1 — Supplementary Information [file 41467_2025_66699_MOESM1_ESM.pdf]

A hierarchy of time constants and reliable signal propagation in the marmoset cerebral cortex

Guanchun Li[1,4], Songting Li[2*], Xiao-Jing Wang[3*]

[1]Courant Institute, New York University, New York, NY 10003.
[2*]School of Mathematical Sciences, MOE-LSC, and Institute of Natural Sciences, Shanghai Jiao Tong University, Shanghai 200240, China.
[3*]Center for Neural Science, New York University, New York, NY 10003.
[4]Present Address: Howard Hughes Medical Institute, Baylor College of Medicine, Houston, TX, 77030, USA.

*Corresponding author(s). E-mail(s): songting@sjtu.edu.cn; xjwang@nyu.edu;
Contributing authors: guanchun.li@nyu.edu;

Supplementary Notes

Mathematical analysis of the relation between FC and SC

In this section, we presented an analysis on the relationship between functional connectivity (FC) and structural connectivity (SC) based on a simplified network model. We assumed the network only has excitatory neurons in each area with symmetric E-to-E connections. Despite this simplification, our analysis is able to conceptually explain the FC-SC relation observed in the original network model.

The dynamics of the $n-$dimensional system are governed by the equation:

$$\frac{d}{dt}\mathbf{r} = -D\mathbf{r} + F\mathbf{r} + I_{\text{ext}},$$

where D is a diagonal matrix representing the intrinsic timescale for each area and F is a symmetric inter-areal connectivity matrix. Both are assumed to be positive-definite matrices. The system can be re-written as:

$$\frac{d}{dt}\mathbf{r} = J\mathbf{r} + I_{\text{ext}} \tag{1}$$

where $J = -D + F$ and $J = J^T$. Notably, all eigenvalues of J must be negative for the system to remain stable.

In the resting state, when each area receives a white noise input of the same variance σ^2 , we presented the following theorem for computing the functional connectivity (FC):

Theorem 1 The covariance matrix of $\mathbf{r}$ denoted by P satisfies the equation:

$$P = -\frac{1}{2}\sigma^2 \left(-D + F\right)^{-1}. \tag{2}$$

Proof Based on the analysis in Deco et al. (2013) [1], the covariance matrix P satisfies the Lyapunov equation:

$$JP + PJ^T + \sigma^2 I_n = 0. \tag{3}$$

Since J is a real symmetric matrix, we can decompose it as

$$J = L\Lambda L^T,$$

where L is a unitary matrix and Λ is a diagonal matrix with all diagonal elements being negative. By left-multiplying and right-multiplying (3) by L^T and L, respectively, we get

$$\Lambda L^T PL + L^T PL\Lambda = -\sigma^2 I_n$$

This leads to

$$L^T PL = -\frac{1}{2}\sigma^2 \Lambda^{-1}.$$

And thus,

$$P = -\frac{1}{2}\sigma^2 L\Lambda^{-1}L^T = -\frac{1}{2}\sigma^2 \left(L\Lambda L^T\right)^{-1} = -\frac{1}{2}\sigma^2 J^{-1}.$$

Substituting $J = -D + F$ back into the equation, we have

$$P = -\frac{1}{2}\sigma^2 \left(-D + F\right)^{-1}.$$

Based on the covariance matrix formula (2), we deduced the relationship between FC and SC as follows:

Theorem 2 When $||D^{-1}F|| < 1$,

$$FC = \frac{1}{2}\sigma^2 D^{-1}\left[SC + R\right] D^{-1}$$

with

$$FC = P, \quad SC = D + F,$$

and the remainder term is:

$$R = D\sum_{k=2}^{\infty}\left(D^{-1}F\right)^k.$$

Proof From Theorem 1, we know that

$$P = -\frac{1}{2}\sigma^2 \left(-D + F\right)^{-1} = \frac{1}{2}\sigma^2 \left(I - D^{-1}F\right)^{-1} D^{-1}$$

Given that $(I - A)^{-1} = I + \sum_{k=1}^{\infty} A^k$ for $||A|| < 1$, we have

$$\begin{aligned} P &= \frac{1}{2}\sigma^2 \left[I + D^{-1}F + \sum_{k=2}^{\infty} \left(D^{-1}F\right)^k\right] D^{-1} \\ &= \frac{1}{2}\sigma^2 D^{-1} \left[D + F + D\sum_{k=2}^{\infty} \left(D^{-1}F\right)^k\right] D^{-1}. \end{aligned}$$

If we assign FC as P and SC as $D + F$ (since it shares the same spatial structure with J), we find that

$$FC = \frac{1}{2}\sigma^2 D^{-1} \left[SC + R\right] D^{-1}$$

with the remainder term:

$$R = D\sum_{k=2}^{\infty} \left(D^{-1}F\right)^k.$$

Theorem 2 suggests that the deviation between FC and SC can be fully encapsulated by the remainder term R. We then sought to understand how the value of R depends on the properties of the weight matrix J. We presented the following theorem:

Theorem 3 $||R|| \to \infty$ as the system (1) approaches critical state, i.e., $\lambda_{\max}(J) \to 0$.

Proof Considering the eigenvalue decomposition of $D^{-1}F$ as $D^{-1}F = V\Psi V^{-1}$ with $\Psi = \mathrm{diag}\left(\psi_1, \psi_2, \cdots, \psi_n\right)$, we know

$$\left(D^{-1}F\right)^k = V\Psi^k V^{-1},$$

and

$$\sum_{k=2}^{\infty} \left(D^{-1}F\right)^k = \sum_{k=2}^{\infty} V\Psi^k V^{-1} = V\left(\sum_{k=2}^{\infty} \Psi^k\right) V^{-1} = VSV^{-1},$$

where

$$S = \mathrm{diag}\left(\frac{\psi_1^2}{1-\psi_1}, \frac{\psi_2^2}{1-\psi_2}, \cdots, \frac{\psi_n^2}{1-\psi_n}\right).$$

Hence we have

$$||R|| = ||D\sum_{k=2}^{\infty} \left(D^{-1}F\right)^k|| \sim O\left(\frac{\psi_*^2}{1-\psi_*}\right)$$

where $\psi_* = \lambda_{\max}\left(D^{-1}F\right)$ is the maximum eigenvalue of $D^{-1}F$. When $\lambda_{\max}(J) \to 0$, based on Lemma 1 below, we can have that $\psi_* = \lambda_{\max}\left(D^{-1}F\right) \to 1$ and thus $||R|| \to \infty$.

Lemma 1 As $\lambda_{\max}(J) \to 0$, it follows that $\lambda_{\max}\left(D^{-1}F\right) \to 1$.

Proof 1. First, we demonstrate that $\lambda_{\max}\left(D^{-1}F\right) < 1$.

Given all eigenvalues of $J = -D + F$ is negative, we know that $D - F$ is a positive definite matrix. Based on the property that for two positive symmetric matrices A, B (Prob.III.6.14; Matrix Analysis, Bhatia 1997):

$$\lambda_{\min}(AB) \geq \lambda_{\min}(A)\,\lambda_{\min}(B).$$

we have

$$\lambda_{\min}\left(D^{-1}[D-F]\right) \geq \lambda_{\min}\left(D^{-1}\right)\lambda_{\min}(D-F) > 0.$$

Since

$$\lambda_{\min}\left(D^{-1}[D-F]\right) = \lambda_{\min}\left(I - D^{-1}F\right) = 1 - \lambda_{\max}\left(D^{-1}F\right),$$

we have

$$\lambda_{\max}\left(D^{-1}F\right) < 1.$$

2. Next, we prove that $D^{-1}F$ contains an eigenvalue approaching 1 as $\lambda_{\max}(J) \to 0$. Letting $\epsilon = \lambda_{\max}(J)$, there exists a corresponding eigenvector $\mathbf{x}$ such that

$$(-D+F)\,\mathbf{x} = \epsilon\mathbf{x},$$

which is equivalent to

$$\left(-I + D^{-1}F\right)\mathbf{x} = \epsilon D^{-1}\mathbf{x},$$

such that

$$D^{-1}F\mathbf{x} = \mathbf{x} + \epsilon D^{-1}\mathbf{x}.$$

As $\lambda_{\max}(J) \to 0$, we know that $\epsilon \to 0$ and

$$D^{-1}F\mathbf{x} \to \mathbf{x};$$

which indicates that $D^{-1}F$ has an eigenvalue approximating 1. Incorporating both findings that $D^{-1}F$ has an eigenvalue near 1 and $\lambda_{\max}\left(D^{-1}F\right) < 1$, we know that

$$\lambda_{\max}\left(D^{-1}F\right) \to 1.$$

Furthermore, the corresponding eigenvector of $D^{-1}F$ also approximates to the eigenvector of J.

If the large entries (the magnitude being substantially nonzero) of the eigenvectors of J associated with near-zero eigenvalues are dense in higher indices (corresponding to areas of higher hierarchy), then the large entries of the remainder R are also densely concentrated within the block of higher indices. This can be shown by the following argument. Let us denote

$$V = (\mathbf{v}_1, \mathbf{v}_2, \cdots, \mathbf{v}_n), \quad V^{-1} = (\mathbf{u}_1, \mathbf{u}_2, \cdots, \mathbf{u}_n)^T.$$

Based on the proof of Theorem 3, the remainder can be expressed as

$$R = DVSV^{-1} = D\sum_{i=1}^{n}\frac{\psi_i^2}{1-\psi_i}\mathbf{v}_i\mathbf{u}_i^T,$$

which gives out the relationship between the remainder and the corresponding eigenmodes of $D^{-1}F$.

Our analysis and numerical results indicate that for the eigenvectors $\mathbf{u}_i, \mathbf{v}_i$ associated with eigenvalues ψ_i close to one, there will be prominent non-zero elements in $\mathbf{u}_i, \mathbf{v}_i$ corresponding to high-level areas. This observation stems from the understanding that the eigenmodes with larger time constants (linked to smaller eigenvalues for $-D+F$, which in turn correspond to eigenvalues closer to 1 for $D^{-1}F$) often involve brain areas with a large gradient of excitation. Those significant non-zero elements in $\mathbf{u}_i, \mathbf{v}_i$ are further amplified by the factor $\psi_i^2/(1-\psi_i)$ (as ψ_i is closer to 1), leading to large entries in the remainder R. This implies a considerable divergence between SC and FC in high-level brian areas. Such findings suggest that in systems exhibiting timescale localization, where higher-level areas possess larger timescales (corresponding to smaller eigenvalues), the discrepancy between SC and FC is more pronounced in these higher-level areas.

Relationship between composite gradient of excitability and timescale of neuron activity

We derive the relationship between $1/(1-h)$ (where h denotes the composite gradient of excitability) and the time constant of neural activity using a simplified version of the orginal model based on the Ornstein–Uhlenbeck process:

$$\tau_E \frac{d}{dt} r_E^i = -r_E^i + \beta_E \left(1 + \eta_E h_i\right) w_{EE} r_E^i + I_{\text{ext,E}}^i \tag{4}$$

Here, the system, comprising both excitatory (E) and inhibitory (I) populations, is approximated by excitatory neurons alone, with the external input modeled as white noise $I_{\text{ext,E}}^i = \tau_E \sigma N(t) \sim N\left(0, \tau_E^2 \sigma^2\right)$.

The auto-correlation function of neural activity satisfying Eq.(4) can be solved as

$$ACF(t) = \frac{\sigma^2}{2\theta_i} e^{-\theta_i t},$$

where

$$\theta_i = \frac{1}{\tau_E}\left(1 - \beta_E w_{EE} - \beta_E \eta_E w_{EE} h_i\right)$$

This yields the timescale of the neural activity as follows

$$\tau_i = \frac{1}{\theta_i} = \tau_E \cdot \frac{1}{1 - \beta_E w_{EE} - \beta_E \eta_E w_{EE} h_i}.$$

Thus, we have:

$$1 - \beta_E w_{EE} - \beta_E \eta_E w_{EE} h_i = \frac{\tau_E}{\tau_i}, \tag{5}$$

and consequently:

$$1 - \beta_E w_{EE} = \beta_E \eta_E w_{EE} h_i + \frac{\tau_E}{\tau_i}. \tag{6}$$

Notably, when the parameters are set in the model, for the high-level area with $h_i \to 1$, the corresponding timescale τ_i is much larger (hundreds of milliseconds) than the intrinsic time constant τ_E (a few milliseconds), resulting in $\tau_E/\tau_i \to 0$. Incorporating this into Eq.(6), we have

$$1 - \beta_E w_{EE} \approx \beta_E \eta_E w_{EE}.$$

Taking this approximation back into Eq.(5), we obtain

$$\beta_E \eta_E w_{EE} (1 - h_i) \approx \frac{\tau_E}{\tau_i},$$

and hence

$$\frac{1}{1 - h_i} \approx \frac{\beta_E \eta_E w_{EE}}{\tau_E} \cdot \tau_i, \tag{7}$$

thereby establishing a linear relationship between $1/(1-h)$ and τ.

Stimulus-dependent timescale of neural activity

This section details our analysis of the network's response following stimulus presentation to a specific brain area, particularly focusing on how the timescale of decaying activity depends on the stimulated area. Through mathematical analysis and numerical examples, we demonstrate that the observed discrepancies in timescales following different stimuli do not reflect a change in intrinsic timescale but rather the interactions among different brain areas.

We consider a linear system governed by the dynamics:

$$\frac{d\mathbf{r}}{dt} = J\mathbf{r}, \quad \mathbf{r}(0) = \mathbf{r}^*, \tag{8}$$

where $\mathbf{r}^*$ represents the system's response post-stimulus, and we denote $t = 0$ as the time when the stimulus is off. Using the eigenvalue decomposition of the weight matrix J, we have

$$J = V\Sigma V^{-1},$$

with eigenvalues $\lambda_1, \lambda_2, \cdots, \lambda_n$ and corresponding eigenvectors $\mathbf{v}_1, \mathbf{v}_2, \cdots \mathbf{v}_n$ where

$$\Sigma = \mathrm{diag}\left(\lambda_1, \lambda_2, \cdots, \lambda_n\right), \quad V = \left(\mathbf{v}_1, \mathbf{v}_2, \cdots \mathbf{v}_n\right) = \left[v_{ik}\right]_{n\times n}.$$

Projecting the firing rate vector $\mathbf{r}$ onto the eigenmode space yields

$$\mathbf{y} = V^{-1}\mathbf{r} = \left(y_1, y_2, \cdots, y_n\right)^T,$$

where each y_i denotes the projection magnitude onto the i-th eigenvector. The dynamics now becomes

$$\frac{d\mathbf{y}}{dt} = \Sigma\mathbf{y}, \quad \mathbf{y}(0) = \mathbf{y}^*, \tag{9}$$

where $\mathbf{y}^* = V^{-1}\mathbf{r}^*$, the initial firing rate profile projected onto the eigen-space.

Solving Eq.(9), we find

$$y_i(t) = y_i^* e^{-\lambda_i t}, \quad \forall 1 \leq i \leq n,$$

which leads to

$$\mathbf{r}(t) = V\mathbf{y}(t) = \sum_{k=1}^{n} y_k(t) \cdot \mathbf{v}_k = \sum_{k=1}^{n} \left(y_k^* e^{-\lambda_k t}\right) \cdot \mathbf{v}_k,$$

and thus

$$r_i(t) = \sum_{k=1}^{n} v_{ik} y_k(t) = \sum_{k=1}^{n} (v_{ik} y_k^*)\, e^{-\lambda_k t}, \quad \forall 1 \leq i \leq n. \tag{10}$$

Eq.(10) illustrates that the dynamics of a given brain area's activity are influenced by individual components with distinct timescales, determined by the term $v_{ik}y_k^*$, which combines the corresponding eigenvector element and the magnitude of $\mathbf{y}^*$ influenced by the response pattern of $\mathbf{r}^*$ post-stimulus.

Timescale localization ensures that V is a sparse matrix, with only a few non-zero elements for each v_{ik} per each given i. This sparsity indicates that each r_i will predominantly be influenced by a limited number of components, leading to distinct timescales across different brain areas. However, a component can still significantly influence the dynamics, even with a small v_{ik}, provided that y_k^* is substantially large. This scenario is particularly likely when certain areas or clusters of areas are intensely activated during stimulus presentation.

Figure S1 illustrates the timescales and the contribution of each eigenmode to various brain areas following stimulus presentation, with either V1 or A4ab as the stimulated brain area. Following stimulation of V1, several eigenmodes associated with low-level visual areas are activated, typically characterized by shorter intrinsic timescales. In contrast, stimulation of A4ab predominantly activates eigenmodes involving a broader range of brain areas, including clusters of high-level areas, which generally exhibit much longer timescales. The activation of these eigenmodes becomes the dominant factor in the system's behavior, significantly influencing the dynamics of low-level brain areas. This predominance explains why even low-level areas exhibit a slower decay in brain activity following stimulation.

In summary, stimulus presentation does not alter the intrinsic component patterns or the timescale of each component. Instead, it modifies the relative contributions of each component to different brain areas. This mechanism accounts for the observed discrepancies in timescales between the resting state and post-stimulus sceanrios. After stimulus presentation, a brain area's timescale is influenced by inputs from other areas or clusters, particularly those strongly activated during the stimulus. This interplay results in the observed variation in decay time constants across different brain regions.

References

[1] Deco, G., Ponce-Alvarez, A., Mantini, D., Romani, G.L., Hagmann, P., Corbetta,

M.: Resting-state functional connectivity emerges from structurally and dynamically shaped slow linear fluctuations. Journal of Neuroscience **33**(27), 11239–11252 (2013)

Supplementary Figures

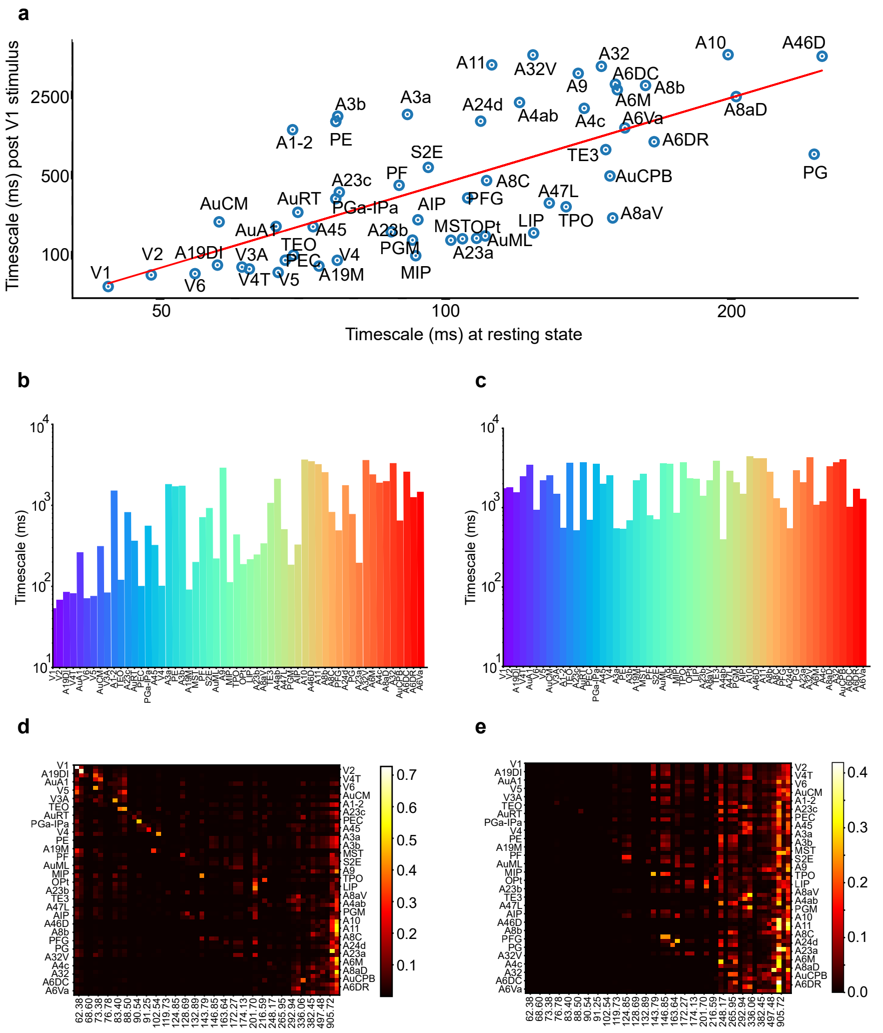

Fig. S1 Stimulus-dependent timescale distribution across areas. (a) Comparison of timescale during the resting state and after V1 stimulus from model simulation. A strong correlation is observed (Pearson $r = 0.69, p = 4.23 \times 10^{-9}$.) (b) The observed timescale gradient following a stimulus to V1, as extracted by fitting to the exponential function. (c) Similar to (b) but with stimulus to A4ab. (d) Relative contribution of each eigenmode to different brain areas (normalized by row) following stimulus presentation to V1. (e) Similar to (d) but with stimulus to A4ab.

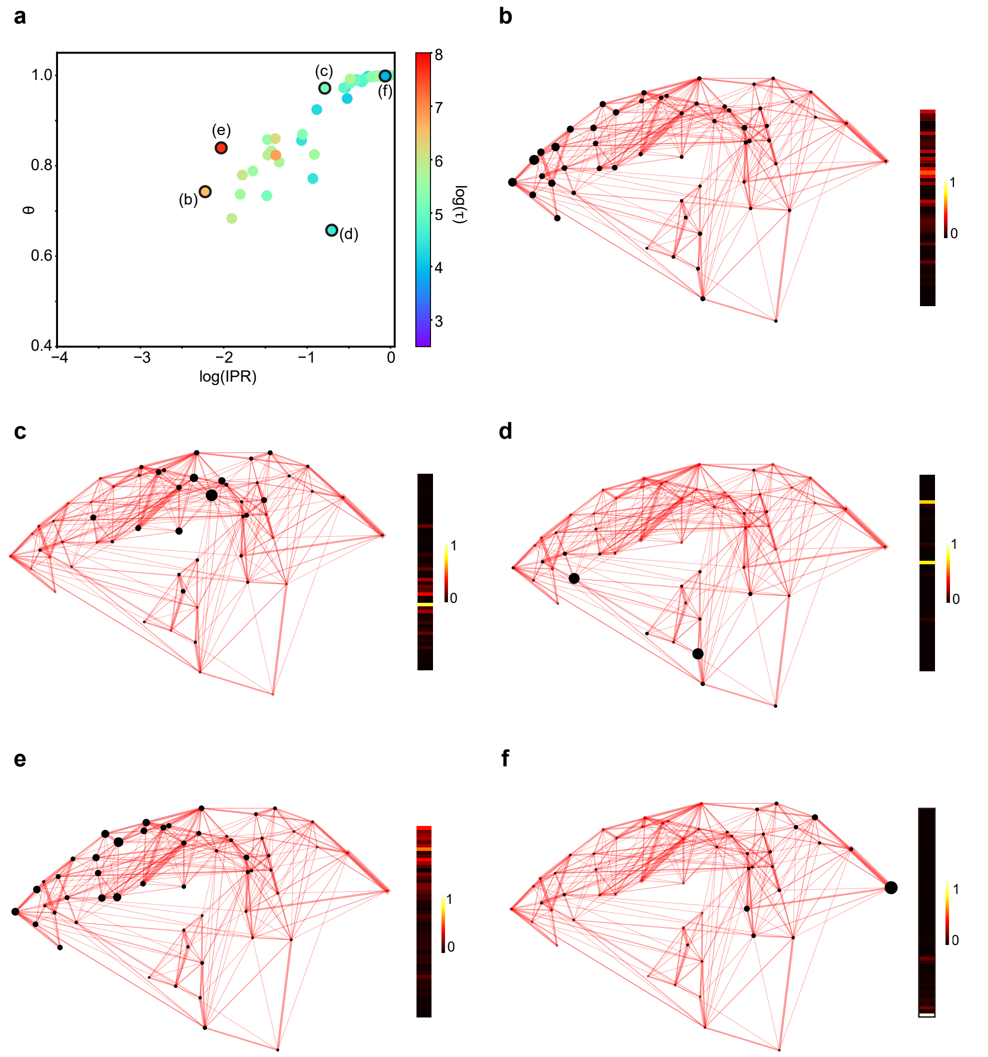

Fig. S2 Two metrics to quantify timescale localization and spatial localization. (a) Scatterplot of Inverse Participation Ratio (IPR) and θ for each eigenmodes of the model system. The color-coded timescale was after taken the logarithm using the natural base e. The five eigenmodes in (b)-(f) are marked with black edges. (b) - (f) Visualization of five representative eigenmodes illustrating timescale localization metrics IPR and θ, with values of eigenmodes plotted aside. The size of each black dot codes the magnitude of the corresponding element in the eigenmode. (b) An eigenmode with both weak timescale localization (IPR $= 0.11$) and weak spatial localization ($\theta = 0.74$). (c) An eigenmode with both strong timescale localization (IPR $= 0.45$) and strong spatial localization ($\theta = 0.97$). (d) An eigenmode with strong timescale localization (IPR $= 0.49$) but relatively weak spatial localization ($\theta = 0.66$). (e) The slowest eigenmode, showing relatively weak timescale localization (IPR $= 0.13$) but relatively strong spatial localization ($\theta = 0.84$). (f) Same as Figure 4c, the fastest eigenmode, showing both strong timescale localization (IPR $= 0.91$) and strong spatial localization ($\theta = 1.00$).

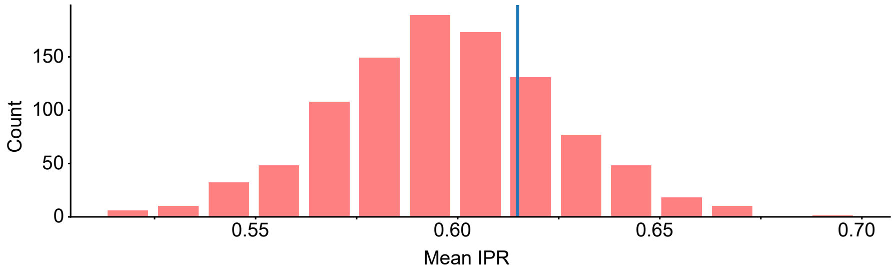

Fig. S3 Impact of shuffled FLNs on timescale localization. The histogram of the degree of timescale localization quantified by the Inverse Participation Ratio (IPR) metric with randomly shuffled FLN. The distribution is close to the value observed in the control condition, as indicated by the vertical line.

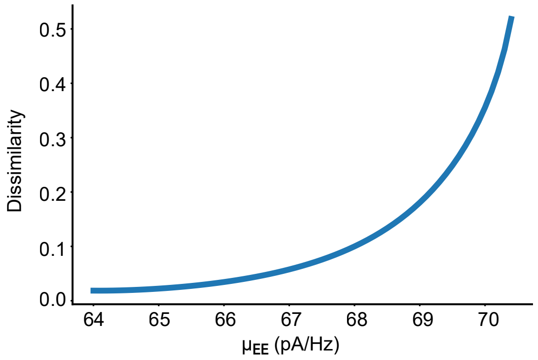

Fig. S4 Dissimilarity between functional connectivity (FC) and structural connectivity (SC) as a function of global coupling strength μ_{EE} of excitatory neurons, another key parameter for criticality. Dissimilarity displays a monotonic increase with the strength of global coupling.

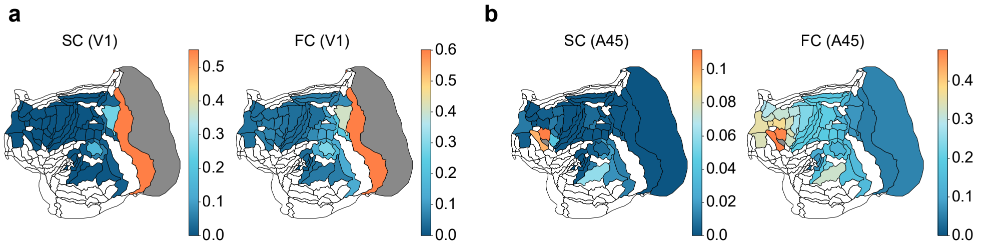

Fig. S5 Comparison of structural connectivity (SC) and functional connectivity (FC) linked to different brain areas in marmoset brain parcellation. Left: Heatmap showing SC (left panel) and FC (right panel) of different brain areas connected to V1. Right: Heatmap showing SC (left panel) and FC (right panel) of different brain areas connected to A45.

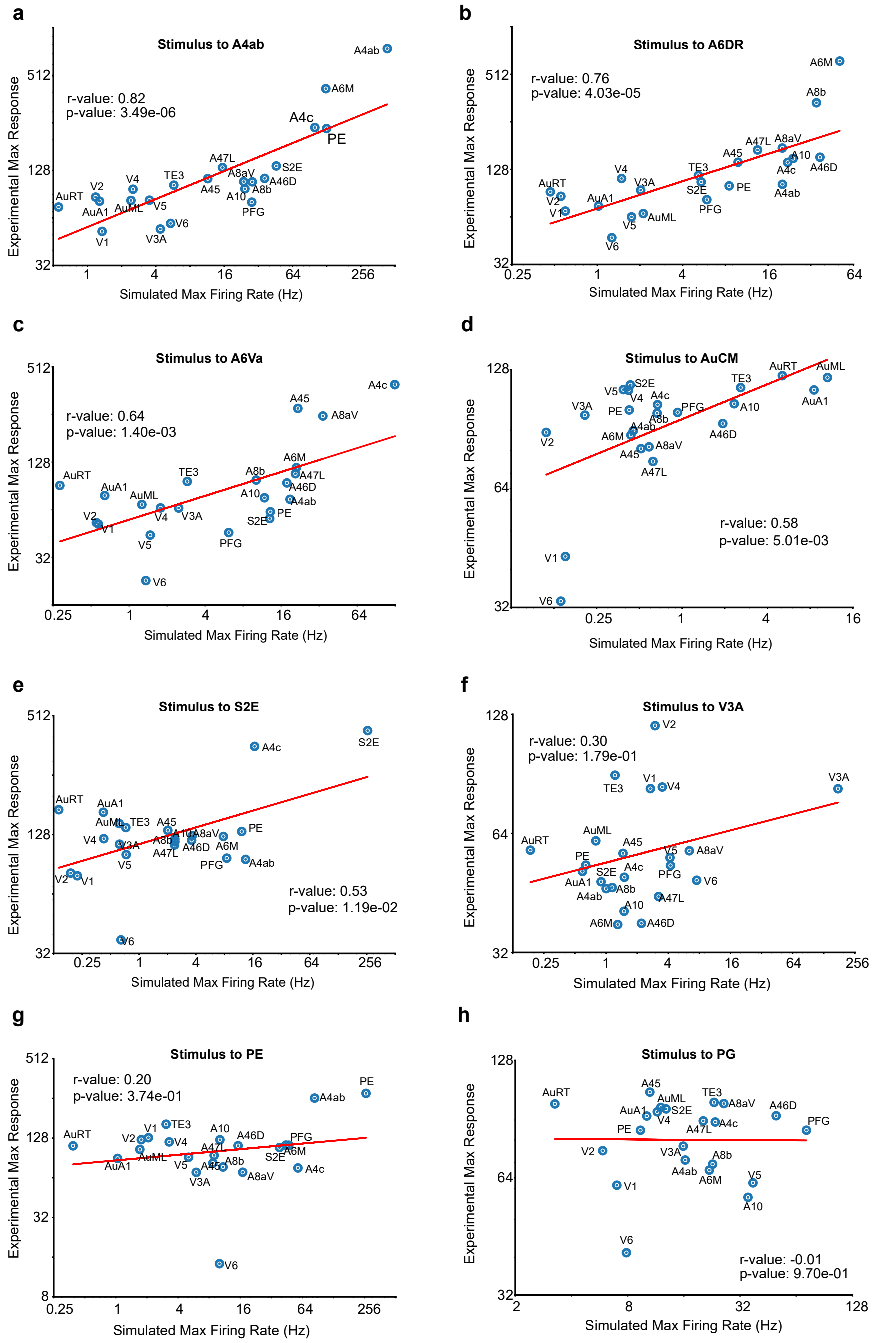

Fig. S6 Comparison of the model's peak response with optogenetics experimental data when different brain areas are stimulated, similar to Figure 7a. (a) - (h) Peak responses of multiple areas after stimulating putative brain area A4ab, A6DR, A6Va, AuCM, S2E, V3A, PE, PG, respectively.

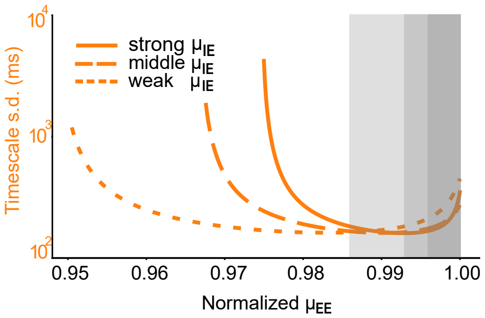

Fig. S7 Impact of global excitatory connectivity (μ_{EE}) on the timescale range, quantified by the standard deviation of timescales of different eigenmodes. The μ_{EE} is normalized to its critical value – the maximum at which the system retains stability.

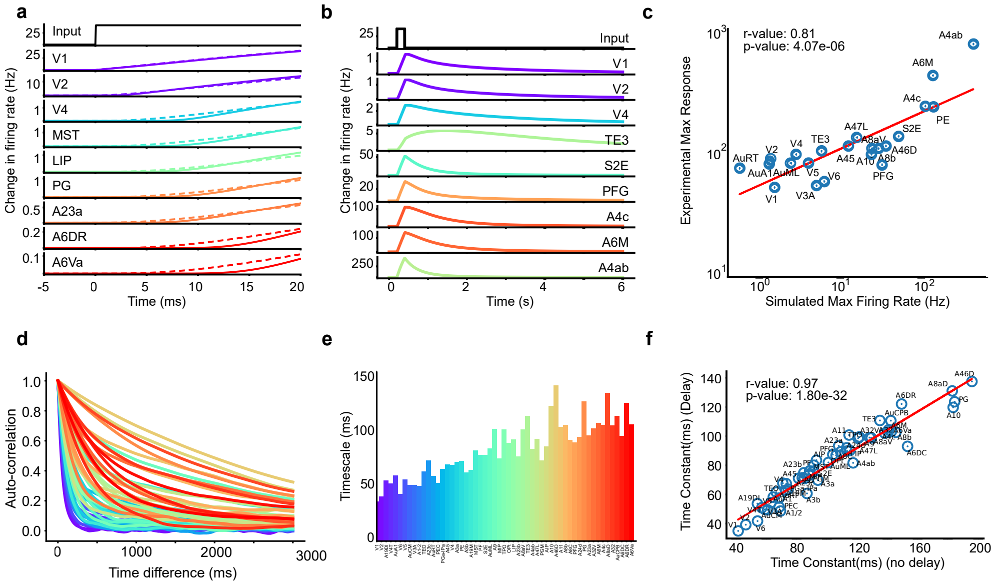

Fig. S8 Results from the *model incorporating explicit axonal conduction delays.* (a) Activity of representative areas following V1 stimulation – focusing the first 20 milliseconds after stimulus onset. Solid line: the activity of model incorporating delays; Dash line: the activity of model without delay. (b) Activity of representative areas within the model incorporating delays following A4ab stimulation. (c) Comparison of the delay-incorporated model's peak response with optogenetics experimental data, demonstrating high model-experimental data consistency (Pearson $r = 0.81, p = 4.57 \times 10^{-6}$). (d) The autocorrelation function of each area's activity in the resting state. (e) Hierarchical timescales extracted from simulated resting state activity of the model incorporating delays. (f) Direct comparison of intrinsic timescales between the delay-incorporated and delay-free models, demonstrating a robust consistence (Pearson $r = 0.97, p = 1.80 \times 10^{-32}$) and indicating minimal impact of conduction delays on the overall timescale hierarchy.

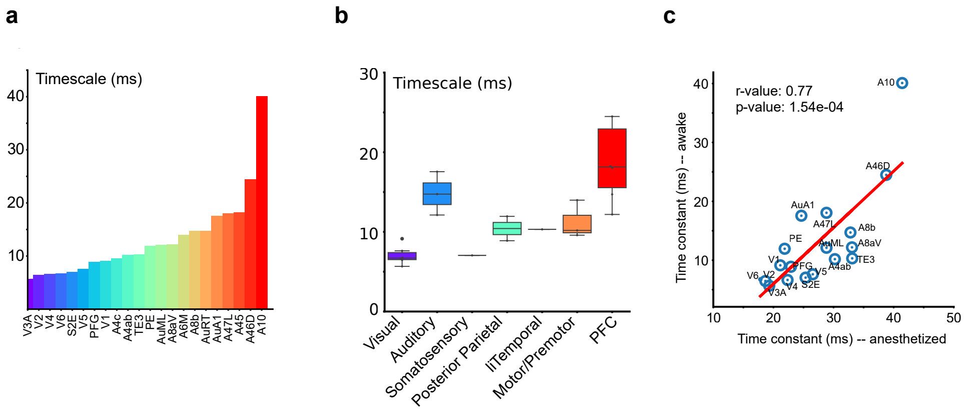

Fig. S9 The timescale hierarchy in the marmoset cortex during the awake resting state. (a) Timescale distribution across the marmoset cortex, derived from neural activity recorded during the awake resting state. Lower-level sensory areas such as V1 and V2 exhibited shorter timescales, in contrast to higher-level areas like A46D and A10 that exhibited longer timescales. (b) Boxplot of timescale statistics for each areal category. Box plots show the media and IQR, with whiskers extending to the minimum and maximum values excluding outliers. Sample sizes (n, number of distinct brain areas per region) from left to right are n =6, 3, 1, 2, 1, 3, and 6. (c) Strong correlation between timescales estimated from anesthetized and awake resting-state data (Pearson $r = 0.77, p = 1.54 \times 10^{-4}$), confirming that the observed timescale hierarchy is consistent across brain states.
