# Supplementary material for: A hierarchy of time constants and reliable signal propagation in the marmoset cerebral cortex
Source: Nat Commun. 2025 Nov 26;16:11640. doi: 10.1038/s41467-025-66699-4 (PMC12749727; doi:10.1038/s41467-025-66699-4)
Supplement: Supplementary file 3 — Transparent Peer Review file [file 41467_2025_66699_MOESM3_ESM.pdf]

nature portfolio

Peer Review File

A hierarchy of time constants and reliable signal propagation in the marmoset cerebral cortex

Corresponding Author: Professor Songting Li

This file contains all reviewer reports in order by version, followed by all author rebuttals in order by version.

Version 0:

Reviewer comments:

Reviewer #1

(Remarks to the Author)
Thank you for inviting me to review this manuscript, in which the authors create a biophysical model of the cerebral cortex in an effort to explain observed variations in the timescale of electrophysiological data collected from the macaque brain.

Comments

* The authors use the term 'cortex' to refer to a particular cortical structure - namely, the cerebral cortex. It is important to disambiguate this from other cortical structures in the brain (such as the cerebellar cortex), which have very different organisational properties.

* The authors treat the cerebral cortex as though it is a disembodied structure, however neuroantomically, the structure is massively (albeit indirectly) connected to a wide range of peripheral (retina, cochlea, spinal cord) and subcortical structures that impose substantial computational constraints on the operation of the cerebral cortex. The assumption that features of the cerebral cortex *alone* are responsible for any observed variance in a measured statistic is an example of a mereological fallacy that should be corrected throughout the text.

* There is a substantial literature evidencing slightly-subcritical dynamics in neural recordings that is completely missing from this paper. Key examples include https://www.nature.com/articles/s41467-018-04725-4, https://pubmed.ncbi.nlm.nih.gov/25788679/, https://www.nature.com/articles/s41467-023-42465-2, https://www.nature.com/articles/ncomms3521, but this list is nowhere near exhaustive. Without the framing imposed by these manuscripts, the paper is unreasonably disconnected from a very relevant literature.

* Did the authors add temporal delays into their model - e.g., proportional to the distance traversed by the estimated white matter connections between regions?

* From the structural connectivity map in Fig 2c, it appears as though there are few (if any) local connections imposed in their model. The data shown in the source material (e.g., Ref #25) do appear to have many local connections, however their figures also appear to be plotted at a finer spatial resolution than the model used in this manuscript. This detail is important, as the density of local connections could change across the same hierarchy observed in this study, and thus may provide an alternative explanation for the observed results.

* The eigenmode/modelling analysis was informative and interesting.

(Remarks on code availability)
The code appears to be well-documented.

Reviewer #2

(Remarks to the Author)
Li et al.'s manuscript "A hierarchy of time constants and reliable signal propagation in marmoset cortex" attempts to ground a multi-area cortex model in experimental ground truth to determine the connectivity between areas that can explain the hierarchy of timescales across the cortical processing hierarchy, and also enable reliable signal propagation. The model leverages marmoset EEG data that allows measurement of the timescale of cortical activity, and anatomical tracing data that includes the number of neurons projecting between the cortical areas. They found that when the network is operating in a state near criticality, both local integration (i.e. timescale hierarchies) and signal propagation are more optimal. Furthermore, the model mechanistically explains why the patterns of structural and functional connectivity are increasingly dissimilar across the cortical hierarchy.
I think that the study is of great interest across systems and computational neuroscience, as it provides a mechanistic explanation for the prominent and systematic changes in activity dynamics (and integration) across the cortical processing hierarchy - and the anatomical basis for the model's connectivity is a major plus. I do have a few comments:
Major
• In the data used to fit the model, the animals were anesthetized with ketamine. I
worry that this could impact activity timescales, as well as the activity states and signal propagation through the network. Perhaps they authors could check in the awake data used for signal propagation that the timescales are still similar in that state?
• Anatomical tracing data is used to determine FLN. Does this also somehow inform the relative interareal 'connection strength' onto E and I cell types, or were these chosen by some other means?
• I think that the authors could make it clearer in places like the results and the methods on what basis the additional composite gradient h is needed (on top of FLN). The increasing number of spines in higher cortex seems to be the reason used. Is the implication then that a given neuron that projects to an area in higher cortex may branch its axons more and form more synapses than a neuron projecting to a 'lower' area? Or is it that more spines = more local recurrence = amplified impact of an input from another area? Further justification and explanation would be helpful in justifying the use of the composite gradient.
Minor
• Some typos:
o line 2 "information process".
o Line137"descried"
o Fig6:"excitability"ismisspelledinafewpanels
• What about cortico-thalamo-cortical connections: can the authors comment on how these could play a role in dynamics and their timescales? Are these more or less captured in the 'mismatch' between correlations and structural connectivity?

• It would be helpful to have more information about the structural data used in this study. Spine counts are used and correlated to the activity timescales - are these total spine counts on entire single neurons, or on specific dendrites? In which layer?
• Fig 6d, e: It is a little confusing for the reader to have the dissimilarity axes in these two panels go from low to high and high to low.

(Remarks on code availability)
I looked at the code - I did not try running it.

Version 1:

Reviewer comments:

Reviewer #1

(Remarks to the Author)
The authors response to my concerns was thorough and authoritative. I have no further reservations.

(Remarks on code availability)

Reviewer #2

(Remarks to the Author)
I appreciate the thorough responses and edits that the authors made, based on my previous comments. I have no further concerns.

(Remarks on code availability)
I reviewed the code in the last round of review, but have not re-checked it.

Open Access This Peer Review File is licensed under a Creative Commons Attribution 4.0 International License, which permits use, sharing, adaptation, distribution and reproduction in any medium or format, as long as you give appropriate credit to the original author(s) and the source, provide a link to the Creative Commons license, and indicate if changes were made.
In cases where reviewers are anonymous, credit should be given to 'Anonymous Referee' and the source.
The images or other third party material in this Peer Review File are included in the article's Creative Commons license, unless indicated otherwise in a credit line to the material. If material is not included in the article's Creative Commons license and your intended use is not permitted by statutory regulation or exceeds the permitted use, you will need to obtain permission directly from the copyright holder.
To view a copy of this license, visit https://creativecommons.org/licenses/by/4.0/

Reviewer #1 (Remarks to the Author):

Thank you for inviting me to review this manuscript, in which the authors create a biophysical model of the cerebral cortex in an effort to explain observed variations in the timescale of electrophysiological data collected from the macaque brain.

Comments

1. The authors use the term 'cortex' to refer to a particular cortical structure - namely, the cerebral cortex. It is important to disambiguate this from other cortical structures in the brain (such as the cerebellar cortex), which have very different organisational properties.

Response: We appreciate the reviewer's comment on the use of the term 'cortex'. Following the reviewer's suggestion, we have replaced the generic term "cortex" with "cerebral cortex" or "neocortex" to avoid ambiguity throughout the entire manuscript—including the title, abstract, main text and figure captions. This change ensures that all references clearly pertain to the cerebral cortex thus it will not be confused with other cortical structures such as the cerebellar cortex.

*2. The authors treat the cerebral cortex as though it is a disembodied structure, however neuroantomically, the structure is massively (albeit indirectly) connected to a wide range of peripheral (retina, cochlea, spinal cord) and subcortical structures that impose substantial computational constraints on the operation of the cerebral cortex. The assumption that features of the cerebral cortex *alone* are responsible for any observed variance in a measured statistic is an example of a mereological fallacy that should be corrected throughout the text.*

Response: We thank the reviewer for pointing out the influence of peripheral and subcortical circuits on cortical computation. We fully agree that the cerebral cortex does not operate in isolation and that peripheral and sub-cortical nuclei impose important computational constraints on cortical dynamics. Our current model uses the cerebral cortical connectome because, to date, the marmoset connectome encompassing the pertinent thalamic, brain-stem, and sensory-peripheral pathways is not available yet at a resolution comparable with the cortical data we use. Incorporating provisional or cross-species estimates of the complete connectome would introduce additional assumptions that cannot be validated empirically. It is worth noting, however, that this work focuses on timescales of spontaneous neural fluctuations, when there is no experimentally designed sensory stimulation to peripheral structures and animals are not engaged in a particular task. Furthermore, the present work and Chaudhuri et al. 2015 previously demonstrate that a hierarchy of time constants can arise in a model of neocortex, as a robust

phenomenon, although its precise pattern is likely to also shaped by interactions with subcortical structures like the thalamus, basal ganglia and cerebellum.

In addition, as shown in Fig. 3c and Fig. 7a, although there are strong correlations between the experimental and modeling results, our model does not perfectly reproduce the timescales and response profiles observed in each area. This discrepancy may stem from the influence of peripheral and subcortical regions, which are not currently included but can be incorporated in future work when a more complete connectome becomes available. To avoid mereological fallacy, we have revised the manuscript throughout (Introduction, lines 2-5, Discussion, lines 530-533) to replace wordings that could be interpreted as attributing observed variance solely to cerebral cortical circuitry. For instance, the opening lines of the **Introduction** (lines 2–5) now read as follows: "The cerebral cortex, together with peripheral sensory organs (e.g., retina, cochlea, spinal cord) and subcortical relays (e.g., thalamus), engages in information processing of external inputs, local integration of signals within regions, and the transmission of these signals along functionally specific pathways." Furthermore, we have revised the relevant sentences in the **Discussion** (lines 530-533) as follows: "This conservation across species emphasizes the robustness of the model framework as a sturdy base for future research probing the relationship between structure and function at a whole-brain level, including the interplay between cortical dynamics and subcortical or peripheral neural systems."

We further include a paragraph in the **Results** "2.2 A multi-regional model of the marmoset neocortex" (page 7, line 172-182) to emphasize that our results describe the contribution of cerebral cortical architecture in the absence of subcortical and peripheral inputs. The paragraph is as follows: "While subcortical structures (e.g., thalamus, brainstem, basal ganglia, cerebellum) and peripheral sensory pathways (e.g., retina, cochlea, spinal cord) exert influences on cerebral cortical dynamics, the present model is restricted to cerebral cortical networks due to the absence of a marmoset-wide, high-resolution subcortical and peripheral connectome. It is worth noting, however, that this work focuses on timescales of spontaneous neural activity during the resting state, when there is no experimentally designed sensory stimulation to peripheral structures and animals are not engaged in a particular task. As future anatomical data allow the integration of subcortical and peripheral circuits, these additional components are expected to improve our model predictions of timescales and signal propagations, with the specific patterns likely influenced by interactions involving subcortical and peripheral structures."

We also added a paragraph in the **Discussion** (page 22, line 586-597) noting the potential contribution of peripheral and subcortical circuits as follows: "While our model focuses exclusively on cortico-cortical connectivity, the cerebral cortex is not anatomically isolated -- subcortical circuits (e.g., thalamus) and peripheral sensory pathways (retina, cochlea, spinal cord) impose important computational constraints on cortical activity. However, due to the lack of a marmoset-wide subcortical connectome at comparable resolution, our model necessarily focused on the corticocortical network, which can later be augmented with subcortical circuits as new data emerge. We anticipate that incorporating cortical-thalamo-cortical (CTC) and peripheral pathways

in future marmoset models will refine our present findings, particularly by enhancing model predictions related to intrinsic timescale patterns, signal propagation, and functional connectivity profiles. Incorporating such pathways represents a key direction for future work as compatible anatomical datasets become available."

References:

1. Chaudhuri, R., Knoblauch, K., Gariel, M. A., Kennedy, H., & Wang, X. J. (2015). A large-scale circuit mechanism for hierarchical dynamical processing in the primate cortex. *Neuron*, *88*(2), 419-431.

3. There is a substantial literature evidencing slightly-subcritical dynamics in neural recordings that is completely missing from this paper. Key examples include https://www.nature.com/articles/s41467-018-04725-4, https://pubmed.ncbi.nlm.nih.gov/25788679/, https://www.nature.com/articles/s41467-023-42465-2, https://www.nature.com/articles/ncomms3521, but this list is nowhere near exhaustive. Without the framing imposed by these manuscripts, the paper is unreasonably disconnected from a very relevant literature.

Response: We thank the reviewer for pointing out these important body of works. We have now integrated them and other relevant literatures explicitly into the **Discussion** (page 20, line 477-495). The new paragraph is as follows:

"Converging evidence across multiple species and brain regions indicates that cortical dynamics operate near a critical point, yet remain slightly subcritical (Petermann et al., 2009; Priesemann et al., 2014; Wilting & Priesemann, 2018). This slight subcriticality is exemplified by a consistent branching ratio of ~0.98 observed in vivo (Wilting & Priesemann, 2018) and truncated neuronal avalanche size distributions in awake cortex (Petermann et al., 2009; Priesemann et al., 2014). Notably, the precise operating point can be modulated by brain states: focused attention shifts cortical activity further into the subcritical state from the near-critical state during resting (Fagerholm et al., 2015), while changes in arousal or anesthesia can also influence the distance from criticality (Munn et al., 2023; Maschke et al., 2024). Operating just below criticality is hypothesized to confer several key functional advantages—ensuring stability against runaway excitation, preserving long-range correlations and large repertoires of activity patterns, enabling efficient information processing across a critical-like broad dynamic range, and allowing flexible tuning of network sensitivity (Moretti & Muñoz, 2013; Priesemann et al., 2014). Theoretically, the cortex's hierarchical modular architecture could yield an extended critical-like regime on the subcritical side (a Griffiths phase) that provides these functional benefits without fine-tuning (Moretti & Muñoz, 2013). Consistent with this "subcriticality" framework, our modeling results for the marmoset cortical demonstrate that a near-critical (slightly subcritical) state supports robust but controlled global signal propagation and naturally extended intrinsic timescales, highlighting

how slight subcriticality effectively balances integration and stability while avoiding the fragility associated with exact criticality."

We hope these additions situate our study within existing literature and address the reviewer's concern about framing.

References:

1. Petermann, T., Thiagarajan, T. C., Lebedev, M. A., Nicolelis, M. A., Chialvo, D. R., & Plenz, D. (2009). Spontaneous cortical activity in awake monkeys composed of neuronal avalanches. *Proceedings of the National Academy of Sciences*, *106*(37), 15921-15926.

2. Priesemann, V., Wibral, M., Valderrama, M., Pröpper, R., Le Van Quyen, M., Geisel, T., ... & Munk, M. H. (2014). Spike avalanches in vivo suggest a driven, slightly subcritical brain state. *Frontiers in systems neuroscience*, *8*, 108.

3. Wilting, J., & Priesemann, V. (2018). Inferring collective dynamical states from widely unobserved systems. *Nature communications*, *9*(1), 2325.

4. Fagerholm, E. D., Lorenz, R., Scott, G., Dinov, M., Hellyer, P. J., Mirzaei, N., ... & Leech, R. (2015). Cascades and cognitive state: focused attention incurs subcritical dynamics. *Journal of Neuroscience*, *35*(11), 4626-4634.

5. Munn, B. R., Müller, E. J., Medel, V., Naismith, S. L., Lizier, J. T., Sanders, R. D., & Shine, J. M. (2023). Neuronal connected burst cascades bridge macroscale adaptive signatures across arousal states. *Nature Communications*, *14*(1), 6846.

6. Maschke, C., O'Byrne, J., Colombo, M. A., Boly, M., Gosseries, O., Laureys, S., ... & Blain-Moraes, S. (2024). Critical dynamics in spontaneous EEG predict anesthetic-induced loss of consciousness and perturbational complexity. *Communications Biology*, *7*(1), 946.

7. Moretti, P., & Muñoz, M. A. (2013). Griffiths phases and the stretching of criticality in brain networks. *Nature communications*, *4*(1), 2521.

4. Did the authors add temporal delays into their model - e.g., proportional to the distance traversed by the estimated white matter connections between regions?

Response: We thank the reviewer for raising this point. Following the reviewer's suggestion, in the revised manuscript, we have extended the model to include explicit axonal-conduction delays both *within and between* cortical areas.

The Implementation is as follows:

i. Inter-areal delays were computed as $\tau_{ij} = d_{ij}/v_{ax}$, where d_{ij} is the measured white-matter tract length between areas i and j (Majka et al. 2020) and a constant axonal conduction velocity $v_{ax} = 3.5\ m/s$ was assumed.

ii. Intra-areal delays were set to 2 ms.

iii. Both the values of axonal conduction velocity and intra-areal delays follow the ones used in our earlier macaque modeling study (Joglekar et al. 2018).

Noticeably, introducing these delays *does not* alter our main conclusions:

i. **Effect on signal propagation.** Figure R1a–c (Supplementary Figure 8a–c) shows that introducing these delays produces the expected tens-of-milliseconds latency of activity onset after stimulus presentation (panel a), yet the overall spatiotemporal pattern, firing-rate magnitude and decay profile — remains virtually unchanged relative to the original delay-free model (compare panel b in Fig. R1 with Fig. 7a, left, in the main text). The delayed model further reproduces the experimentally measured population response with a high correlation (panel c).

ii. **Effect on resting-state temporal hierarchy,** Figure R1d–f (Supplementary Figure 8d-f)**.** During spontaneous activity, cortical areas still exhibit similar autocorrelation decay profiles (panel d) and a robust timescale hierarchy (panel e). Time constants derived from the delayed model and the original model are highly correlated (r = 0.97; panel f), indicating that the principal conclusions regarding localization of intrinsic timescales are insensitive to the addition of biologically realistic conduction lags.

All modelling choices and equations have been added to "Methods" (page 25-26, line 686-710) as the subsection "4.4 Incorporation of axonal conduction delays," and the model results shown in Fig. R1 are provided in Supplementary Figure S8 (page 46). We explicitly referenced these results within the main text, specifically in the Results "2.3 Emergence of timescale hierarchy in the marmoset network model" (page 9, lines 206-208): "Moreover, the timescale gradient and its close consistency with experimental data remain robust even when biologically realistic axonal conduction delays are incorporated into the model (Figure S8)." Similarly, in Results "2.6 Signal propagation in the near-critical state" (page 16, lines 403-405), we note: "Similarly, this strong agreement with experiments was conserved after incorporating biologically realistic axonal conduction delays into our model (Figure S8)." We hope these additions have addressed the reviewer's query about temporal delays.

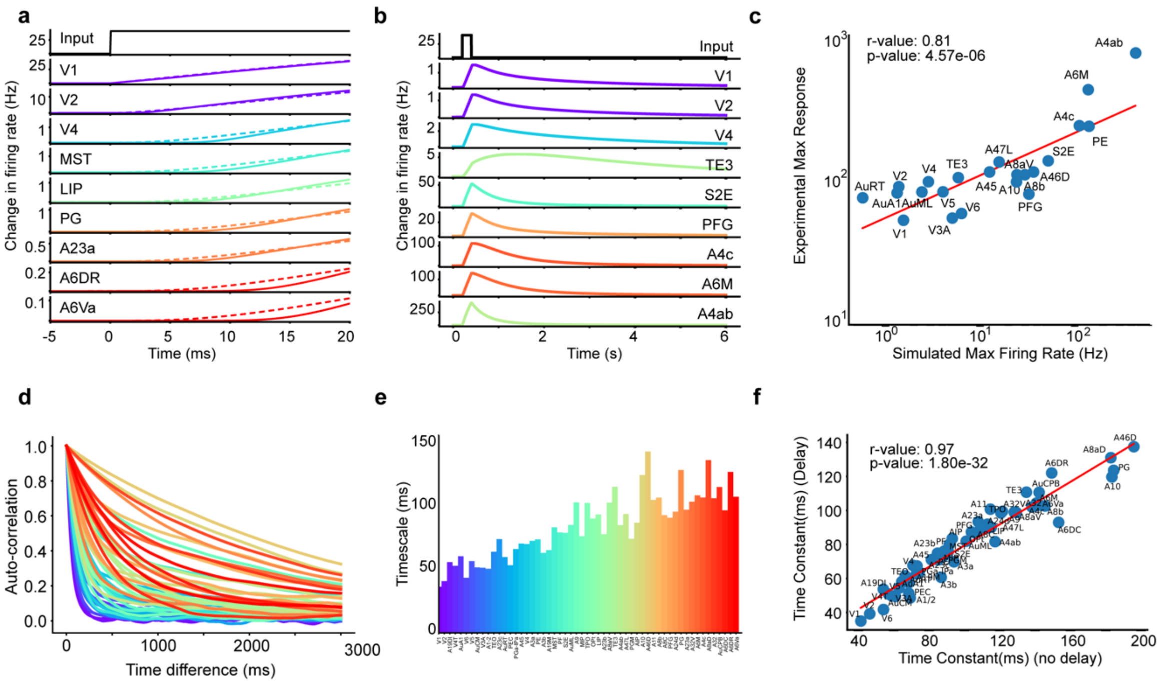

Fig. R1. Results from the *model incorporating axonal conduction delays.* (a) Activity of representative areas following V1 stimulation – focusing the first 20 milliseconds after stimulus onset. Solid line: the activity of model incorporating delays; Dash line: the activity of model without delay. (b) Activity of representative areas in the model incorporating delays following A4ab stimulation. (c) Comparison of the delay-incorporated model's peak response with optogenetics experimental data, demonstrating high model-experimental data consistency ($r = 0.81, p = 4.57 \times 10^{-6}$). (d) The autocorrelation function of each area's activity in the resting state. (e) Hierarchical timescales extracted from simulated resting state activity of the model incorporating delays. (f) Direct comparison of intrinsic timescales between the delay-incorporated and delay-free models, demonstrating a robust consistence ($r = 0.97, p = 1.80 \times 10^{-32}$) and indicating minimal impact of conduction delays on the overall timescale hierarchy.

References:

1. Majka, P., Bai, S., Bakola, S., Bednarek, S., Chan, J. M., Jermakow, N., ... & Rosa, M. G. (2020). Open access resource for cellular-resolution analyses of corticocortical connectivity in the marmoset monkey. *Nature communications*, 11(1), 1133.

2. Joglekar, M. R., Mejias, J. F., Yang, G. R., & Wang, X. J. (2018). Inter-areal balanced amplification enhances signal propagation in a large-scale circuit model of the primate cortex. *Neuron*, 98(1), 222-234.

5. From the structural connectivity map in Fig 2c, it appears as though there are few (if any) local connections imposed in their model. The data shown in the source material (e.g., Ref #25) do appear to have many local connections, however their figures also appear to be plotted at a finer spatial resolution than the model used in this manuscript. This detail is important, as the density

of local connections could change across the same hierarchy observed in this study, and thus may provide an alternative explanation for the observed results.

Response: We apologize for the confusion created by Fig 2c. For visual clarity we displayed only inter-areal projections in Fig 2c; all intra-areal (local) synapses were deliberately omitted in the plot but are fully implemented in the model that drives every result in the paper. Specifically, each cortical area comprises excitatory and inhibitory populations connected by four local weights within each area: $w_{EE}, w_{EI}, w_{IE}, w_{II}$ (see Page 24, Equation (1)-(2), in **Methods**, "4.3 Multi-regional cortical model architecture" and Fig 2b).

Consistent with the reviewer's comment, the density of local connections indeed changes across the same hierarchy. Because our model adopts an areal resolution that aggregates neurons within each area measured in (Ref 25 Majka et al. 2020) as a single area node, the density of local connections is effectively captured by the local connection weights. Accordingly, the same composite excitatory gradient that defines the inter-areal hierarchy is also applied to these local weights in the model**.** Thus, *local recurrent strength increases systematically from sensory to association areas*, which do indeed contribute to the observed temporal gradients. We have added this in the revised caption of Fig 2c for the clarification: "Only inter-areal (long-range) projections are shown for clarity." We hope this clarification, together with the explicit reference to the math formulations, addresses the reviewer's concern.

References:

1. Majka, P., Bai, S., Bakola, S., Bednarek, S., Chan, J. M., Jermakow, N., ... & Rosa, M. G. (2020). Open access resource for cellular-resolution analyses of corticocortical connectivity in the marmoset monkey. *Nature communications*, 11(1), 1133.

6. The eigenmode/modelling analysis was informative and interesting.

Response: We thank the reviewer for the acknowledgment.

Reviewer #1 (Remarks on code availability):

The code appears to be well-documented.

Response: We thank the reviewer for the acknowledgment.

Reviewer #2 (Remarks to the Author):

Li et al.'s manuscript "A hierarchy of time constants and reliable signal propagation in marmoset cortex" attempts to ground a multi-area cortex model in experimental ground truth to determine the connectivity between areas that can explain the hierarchy of timescales across the cortical processing hierarchy, and also enable reliable signal propagation. The model leverages marmoset EEG data that allows measurement of the timescale of cortical activity, and anatomical tracing data that includes the number of neurons projecting between the cortical areas. They found that when the network is operating in a state near criticality, both local integration (i.e. timescale hierarchies) and signal propagation are more optimal. Furthermore, the model mechanistically explains why the patterns of structural and functional connectivity are increasingly dissimilar across the cortical hierarchy.

I think that the study is of great interest across systems and computational neuroscience, as it provides a mechanistic explanation for the prominent and systematic changes in activity dynamics (and integration) across the cortical processing hierarchy - and the anatomical basis for the model's connectivity is a major plus. I do have a few comments:

Major

1. *In the data used to fit the model, the animals were anesthetized with ketamine. I worry that this could impact activity timescales, as well as the activity states and signal propagation through the network. Perhaps they authors could check in the awake data used for signal propagation that the timescales are still similar in that state?*

Response: We share the reviewer's concern and therefore repeated the timescale analysis *in the awake data used for signal propagation*. We extracted the first 5 seconds of the session — prior to any stimulation—to obtain a resting-state segment free of task or laser effects. Using the same spectral method as for the *anesthetized* data, we obtain a clear hierarchy of intrinsic timescales across areas (Fig. R2a) that closely mirrors the *anesthetized* data. When areas are grouped by categories, timescales again increase from low-level sensory to high-level association areas (Fig. R2b). Directly comparing the time constants of neural activity in the awake state with those in the anesthetized state yields a strong correlation of $r = 0.77$ (Fig. R2c), confirming that the relative ordering of timescales is approximately preserved.

Interestingly, we also note that the absolute values of the timescales are slightly lower in the awake state. Two factors likely contribute: (i) wakefulness reduces slow, anesthesia-related oscillations, leading to faster decorrelation of neural activity (Sellers et al. 2015), and (ii) the shorter duration of the awake recording ($5s$ vs ~$35s$) likely underestimates the time constants. In summary, these

findings suggest that the timescale hierarchy derived from the *anaesthetized* dataset is robust across behavioral states.

We have now updated the subsection "2.1 Experimental evidence of timescale hierarchy in the marmoset neocortex" in **Results** (pages 4-5, line 129-133) summarizing the analysis results from the awake resting state data. The sentences are as follows: "Furthermore, by analyzing the first 5 seconds of awake resting-state recordings [36], we observed a clear intrinsic timescale hierarchy across cortical areas closely matching the anesthetized state (Figure S9a-b), with a strong correlation confirming consistency in the relative ordering of timescales (Figure S9c)." All data processing and analysis details have been added into **Methods** (page 23, line 627-633) under the updated subsection "4.1 Experimental data sources," and the newly obtained awake-state timescale results (Figure R2) are included as Figure S9 in the **Supplementary materials** (page 47). We hope these additions have addressed the reviewer's query regarding timescale during the awake resting state.

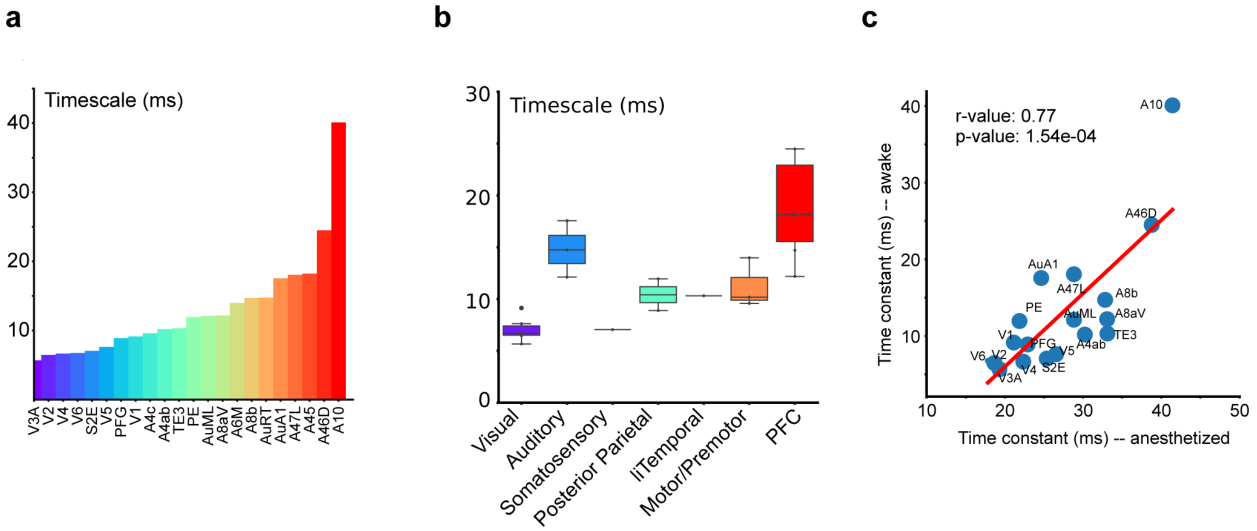

Fig. R2. The timescale hierarchy in the marmoset cortex during the *awake resting state*. (a) Timescale distribution across the marmoset cortex, derived from neural activity recorded during the awake resting state. Lower-level sensory areas such as V1 and V2 exhibited shorter timescales, in contrast to higher-level areas like A46D and A10 that exhibited longer timescales. (b) Boxplot of timescale statistics for each areal category. Box plots show the media and IQR, with whiskers extending to the minimum and maximum values excluding outliers. (c) Strong correlation between timescales estimated from anesthetized and awake resting-state data ($r = 0.77, p = 1.54 \times 10^{-4}$), confirming that the observed timescale hierarchy is consistent across brain states.

References:

1. Sellers, K. K., Bennett, D. V., Hutt, A., Williams, J. H., & Fröhlich, F. (2015). Awake vs. anesthetized: layer-specific sensory processing in visual cortex and functional connectivity between cortical areas. *Journal of neurophysiology*, 113(10), 3798-3815.

2. *Anatomical tracing data is used to determine FLN. Does this also somehow inform the relative interareal 'connection strength' onto E and I cell types, or were these chosen by some other means?*

Response: We thank the reviewer for the important question. The fraction-of-labelled-neurons (FLN) metric we use -- derived from retrograde tracer studies (Majka et al., 2020; Theodoni et al., 2022) represent the fraction of all neurons in source area j that project to target area i. These tracers do not distinguish the neurotransmitter identity of the labelled neurons, and the published datasets therefore provide no direct information about whether the postsynaptic targets in the recipient area are excitatory (E) or inhibitory (I) cells.

In our model we therefore treat FLN as the density of *long-range excitatory* axons and assume that these axons innervate both local E and I populations with identical topography but different synaptic efficacy -- hence we apply the same FLN matrix to both cell classes. The relative efficacy of inter-areal input onto E versus I populations is controlled by two global scaling parameters, μ_{EE} and μ_{IE}. To be specific, for every pair of areas $j \rightarrow i$, the weight W_{ij} derived from FLN is multiplied by a global gain μ_{EE} when the postsynaptic target is excitatory and by μ_{IE} when the target is inhibitory. The two gain parameters were fine-tuned so that the resulting large-scale circuit operates near criticality while jointly reproducing (i) the observed hierarchy of intrinsic timescales during the resting state and (ii) the amplitude of signal propagation across areas evoked by transient stimulation. Once μ_{EE} and μ_{IE} were fixed by this procedure, they were held constant for all simulations reported in the manuscript.

We acknowledge that cell-type–resolved tract-tracing data would allow a more mechanistic treatment of inter-areal E/I specificity. As such datasets become available in the future, our framework can be updated by replacing the global scalars μ_{EE} and μ_{IE} with projection-specific ratios. We hope this clarifies how the FLN data were used and how relative E- and I-targeted connection strengths were assigned in the model.

We have now added a new paragraph in **Discussion** (page 22, line 573-585) explicitly clarifying the rationale behind setting relative inter-areal connection strengths onto E and I cell types. The new paragraph is as follows: "Another important consideration relates to the anatomical connectivity data used: the tracer-derived FLN weights currently employed in our large-scale marmoset cortical model do not specify neurotransmitter identity of target neurons (Majka et al., 2020; Theodoni et al., 2022). We therefore applied an identical FLN connectivity matrix to both excitatory (E) and inhibitory (I) neuronal populations within each cortical region, with their relative efficacies modulated by two global parameters, μ_{EE} and μ_{IE}. These global scaling parameters were carefully tuned to simultaneously satisfy crucial dynamical constraints—notably, the observed hierarchy of intrinsic timescales and robust inter-areal signal propagation—and were subsequently kept fixed throughout all simulations. While this simplification enables effective modeling of large-scale cortical dynamics, it neglects potential heterogeneity in cell-type specific projections. Future availability of cell-type resolved anatomical tracing data will refine the model by introducing projection-specific E/I targeting ratios instead of global scalar parameters."

References:

1. Majka, P., Bai, S., Bakola, S., Bednarek, S., Chan, J. M., Jermakow, N., ... & Rosa, M. G. (2020). Open access resource for cellular-resolution analyses of corticocortical connectivity in the marmoset monkey. *Nature communications*, 11(1), 1133.

2. Theodoni, P., Majka, P., Reser, D. H., Wójcik, D. K., Rosa, M. G., & Wang, X. J. (2022). Structural attributes and principles of the neocortical connectome in the marmoset monkey. *Cerebral Cortex*, 32(1), 15-28.

3. *I think that the authors could make it clearer in places like the results and the methods on what basis the additional composite gradient h is needed (on top of FLN). The increasing number of spines in higher cortex seems to be the reason used. Is the implication then that a given neuron that projects to an area in higher cortex may branch its axons more and form more synapses than a neuron projecting to a 'lower' area? Or is it that more spines = more local recurrence = amplified impact of an input from another area? Further justification and explanation would be helpful in justifying the use of the composite gradient.*

Response: We thank the reviewer for providing us with the opportunity to clarify the rationale behind the composite gradient h. We would like to point out that the FLN matrix only describes the *fraction* of labelled neurons projecting from source area j to target area i. To be more specific, FLN is a *row-normalized* measure, and the row sum of FLN satisfies: $\sum_{j \neq i} FLN_{ij} = 1$ for every target area i. Consequently, FLN does not contain information about the *absolute* number of long-range synapses each target area receives. The total long-range synapse number of a neuron in each target area can be reflected in the composite gradient, which highly correlates with spine count gradient based on anatomical studies below:

(i) The spine counts on the dendrites of pyramidal neurons, rise monotonically from lower-order sensory cortex to higher-order associative cortex (Theodoni et al. 2022), implying that single neurons in higher-order areas physically possess more excitatory synapses. Thus, along the sensory-to-association hierarchy, the total synaptic input number increases.

(ii) Markov *et al.* (2011; Fig. 2A) further show that, for every area examined, ~80 % of its labelled inputs are intrinsic (local) and ~20 % are extrinsic (from other areas). Because this proportion is essentially constant across the cortex, a larger total synaptic input number at higher-order areas directly implies a larger *absolute* number of both local recurrent and long-range synapses—even though their *fraction* remains unchanged.

Taken together these findings indicate that neurons in higher-order areas receive a larger number of *total* excitatory synaptic inputs — both long-range and local inputs —than neurons in lower areas. The composite gradient h therefore re-introduces this biologically motivated scaling by multiplying all excitatory weights by a factor proportional to the spine-count gradient. Importantly,

the gradient does *not* assume extra axonal branching in a single projection, nor does it selectively boost local recurrence; rather, it captures the larger pool of postsynaptic spines available to receive the inputs in higher order areas, which amplifies the functional impact of any incoming drive.

We have now added a new paragraph in **Methods** (4.3 “Multi-regional cortical model architecture”, page 24-25, line 664-680) to clarify the motivation and rationale underlying the composite gradient. The new paragraph is as follows:

“To complement our large-scale model that uses FLN to describe inter-areal connectivity, we additionally introduced a composite gradient of excitation, h. Because the FLN matrix is normalized per target area (each row sums to 1), it encodes only relative inter-areal connectivity but does not capture the area-wise differences in the absolute number of synapse inputs received by each target area. Anatomical evidence indicates that neurons in higher-order association cortex receive more inputs per cell than those in early sensory areas. For instance, dendritic spine counts increase systematically from sensory to association areas (Theodoni et al., 2022). Furthermore, a uniform ~80%/20% division of local versus long-range inputs across areas (Markov et al., 2011) implies that higher-order areas inherently possess greater numbers of both local and inter-areal synapses. Thus, neurons in association cortex receive a larger overall drive than those in lower areas. To account for this gradient, we define a composite gradient of excitation h as an area-specific scaling factor applied to all synaptic weights, effectively representing the increasing synaptic density in higher cortical areas while preserving the empirically measured FLN connectivity pattern. Within our model, h_i is a factor scaling both local (intra-areal) and long-range (inter-areal) excitatory inputs and varies across different areas.”

References:

1. Theodoni, P., Majka, P., Reser, D. H., Wójcik, D. K., Rosa, M. G., & Wang, X. J. (2022). Structural attributes and principles of the neocortical connectome in the marmoset monkey. *Cerebral Cortex*, 32(1), 15-28.

2. Markov, N. T., Misery, P., Falchier, A., Lamy, C., Vezoli, J., Quilodran, R., ... & Knoblauch, K. (2011). Weight consistency specifies regularities of macaque cortical networks. *Cerebral cortex*, 21(6), 1254-1272.

Minor

4. *Some typos:*
o *line 2 "information process".*
o *Line137"descried"*
o *Fig6:"excitability"is misspelled in a few panels*

Response: We thank the reviewer for pointing out these typographical errors. We have carefully reviewed and corrected all the mentioned typos.

5. *What about cortico-thalamo-cortical connections: can the authors comment on how these could play a role in dynamics and their timescales? Are these more or less captured in the 'mismatch' between correlations and structural connectivity?*

Response: We agree with the reviewer that cortical-thalamo-cortical (CTC) loops are likely to modulate both propagation dynamics and intrinsic timescales and could partly account for part of the "structure–function mismatch" reported in our manuscript. A potential evidence has been shown in the large-scale mouse model of Ding *et al. (*2024), where including CTC loops helped sustain distributed persistent activity. Nonetheless, they also showed that several cortical dynamical signatures—such as whether an area displays persistent firing and the absolute firing-rate range—remain qualitatively similar when the thalamus is omitted. This finding suggests that the cortical connectomes captured in our present model sets the *baseline* temporal structure, whereas CTC loops may further stabilize activity and enhance inter-areal correlation.

We therefore predict that incorporating marmoset CTC loops would *refine*, rather than overturn, our main findings: (i) it would modulate the mismatch between functional correlations and corticocortical weights, and (ii) it would slightly lengthen the intrinsic timescales of higher-order areas by providing an additional reverberatory pathway. Because a high-resolution marmoset-wide dataset that links each cortical area to its thalamic partnersis not available yet, in this work we are only able to analyze the cerebral cortex-only network first and we explicitly flagged the limitations of not including CTC in the model.

We therefore have added a paragraph in the Discussion (page 22, line 586-597) noting the potential contribution of CTC loops as follows: "While our model focuses exclusively on cortico-cortical connectivity, the cerebral cortex is not anatomically isolated -- subcortical circuits (e.g., thalamus) and peripheral sensory pathways (retina, cochlea, spinal cord) impose important computational constraints on cortical activity. However, due to the lack of a marmoset-wide subcortical connectome at comparable resolution, our model necessarily focused on the corticocortical network, which can later be augmented with subcortical circuits as new data emerge. We anticipate that incorporating cortical-thalamo-cortical (CTC) and peripheral pathways in future marmoset models will refine our present findings, particularly by enhancing model predictions related to intrinsic timescale patterns, signal propagation, and functional connectivity profiles. Incorporating such pathways represents a key direction for future work as compatible anatomical datasets become available."

References:

1. Ding, X., Froudist-Walsh, S., Jaramillo, J., Jiang, J., & Wang, X. J. (2024). Cell type-specific connectome predicts distributed working memory activity in the mouse brain. *elife*, *13*, e85442.

6. *It would be helpful to have more information about the structural data used in this study. Spine*

counts are used and correlated to the activity timescales - are these total spine counts on entire single neurons, or on specific dendrites? In which layer?

Response: We thank the reviewer's comments, which provides us the opportunity to clarify the structural dataset. The spine values used in Fig. 2 and 3 correspond to the *mean basal-dendrite spine counts of layer-3 pyramidal neurons* in each cortical area. These measurements were collected from several studies and collated in Supplementary Table 4 of "*Structural Attributes and Principles of the Neocortical Connectome in the Marmoset Monkey*" (Theodoni et al. 2022). To make this provenance explicit, we have added the following statement in the revised caption of Fig. 2f: "Layer-3 pyramidal-cell spine counts were taken from the cross-area compilation in Supplementary Table 4 of [Theodoni et al. 2022]; sampling details are given in the cited source articles." We hope these additions can give readers a clear picture of how the spine-count variable was defined and measured.

References:

1. Theodoni, P., Majka, P., Reser, D. H., Wójcik, D. K., Rosa, M. G., & Wang, X. J. (2022). Structural attributes and principles of the neocortical connectome in the marmoset monkey. *Cerebral Cortex*, 32(1), 15-28.

7. *Fig 6d, e: It is a little confusing for the reader to have the dissimilarity axes in these two panels go from low to high and high to low.*

Response: We thank the reviewer for this suggestion. To avoid any confusion, we have re-oriented the dissimilarity axis in Fig. 6e so that both panels now display "low → high" from left to right. The figure and caption have been updated in the revised manuscript. The updated Figure 6e is as follows:

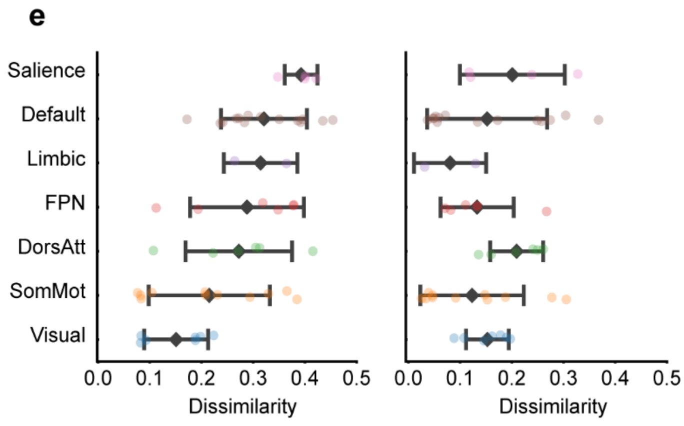

New Figure 6 (e): FC-SC dissimilarity across seven functional subnetworks of the marmoset brain. Left: the control model result. Right: The model result with gradient removed.

Reviewer #2 (Remarks on code availability):

I looked at the code - I did not try running it

Response: We thank the reviewer for inspecting the repository.

Reviewer #1 (Remarks to the Author):

The authors response to my concerns was thorough and authoritative. I have no further reservations.

Response: We thank the reviewer for the thoughtful feedback and are pleased that our revisions addressed all concerns.

Reviewer #2 (Remarks to the Author):

I appreciate the thorough responses and edits that the authors made, based on my previous comments. I have no further concerns.

Response: We thank the reviewer for the positive assessment and helpful suggestions throughout the review process.

Reviewer #2 (Remarks on code availability):

I reviewed the code in the last round of review, but have not re-checked it.

Response: We appreciate the reviewer’s previous review of the code.
